# Editorial: Lignocellulosic biomass-based materials: Design, fabrication, and applications

**DOI:** 10.3389/fbioe.2023.1188168

**Published:** 2023-03-24

**Authors:** Han-Min Wang, Weijun Yang, Mika H. Sipponen, Lin Dai

**Affiliations:** ^1^ State Key Laboratory of Biobased Fiber Manufacturing Technology, Tianjin Key Laboratory of Pulp and Paper, China Light Industry Key Laboratory of Papermaking and Biorefinery, Tianjin University of Science and Technology, Tianjin, China; ^2^ The Key Laboratory of Synthetic and Biological Colloids, Ministry of Education, School of Chemical and Material Engineering, Jiangnan University, Wuxi, China; ^3^ Department of Materials and Environmental Chemistry, Stockholm University, Stockholm, Sweden; ^4^ Wallenberg Wood Science Center, Department of Materials and Environmental Chemistry, Stockholm University, Stockholm, Sweden

**Keywords:** cellulose, hemicellulose, lignin, biomass conversion, materials

Lignocellulosic biomass represents the primary renewable resource for bio-based materials and chemicals, which also exhibits excellent potential as alternatives to fossil-based materials and energy owing to their desirable biodegradability, sustainability, and unique structural characteristics. Benefiting from these admirable advantages, lignocellulosic biomass-based materials has been a rapidly growing research field in the past few decades. Owing to the increased efforts and interest in this direction, pivotal progress has been achieved in harnessing the unique properties and functionalities of cellulose, hemicelluloses, and lignin. In addition, extractives and other minor components of lignocellulosic biomass are increasingly recognized as functional components of bio-based materials.

**FIGURE 1:**
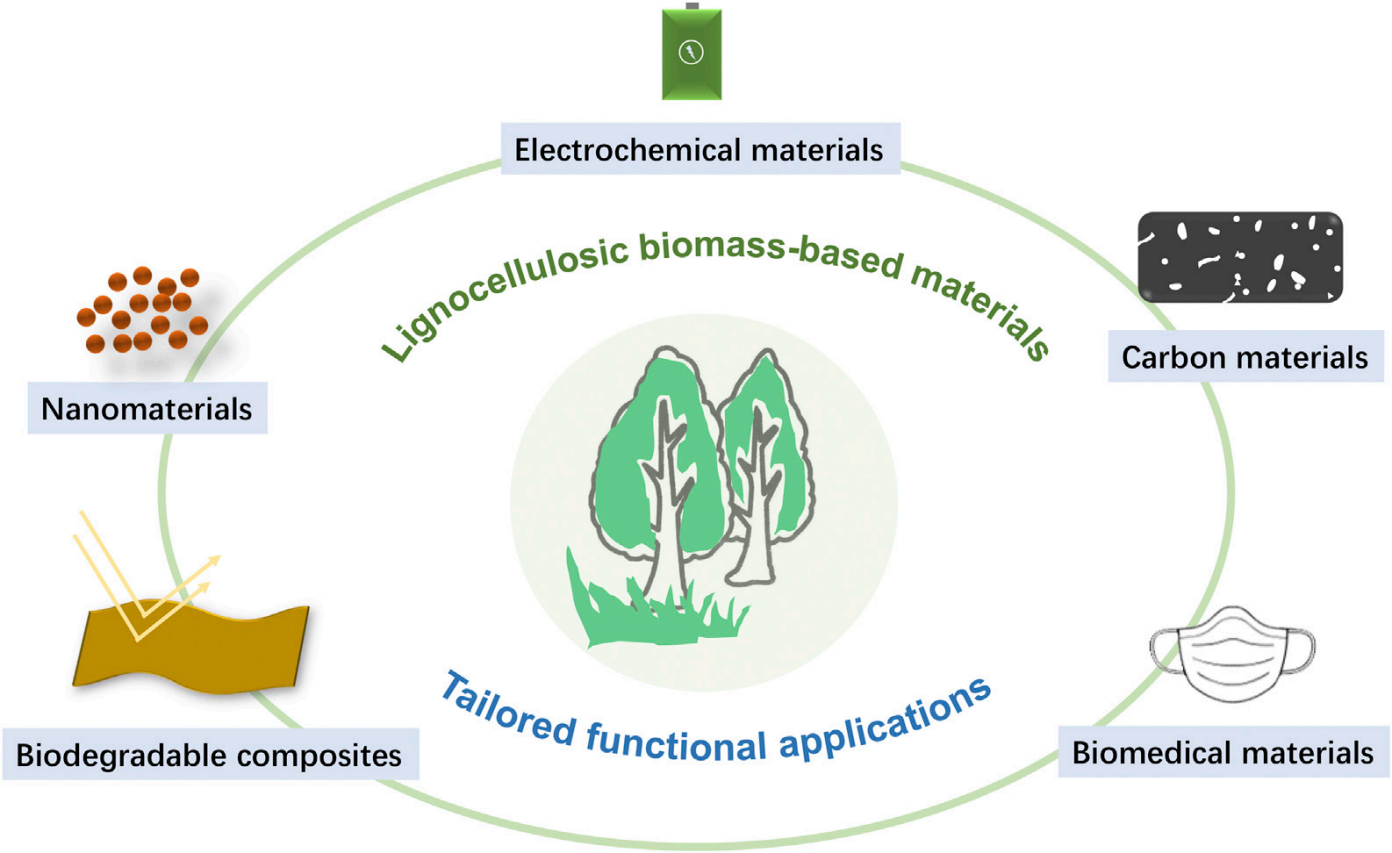
Tailored biobased materials from lignocellulosic biomass.

Fossil-based plastics are cheap and durable materials, broadly applied in various fields, for example, packaging, electronics, and aviation. Nevertheless, the timescale of their complete biodegradation varies from hundreds to even thousands of years due to their stable long polymer chains. As a consequence, there will be over 11 billion metric tons of plastics accumulating in the natural environment by 2025, causing severe environmental pollution. Modern lignocellulosic biorefineries are greatly extending the design and fabrication of biomass-based materials toward a fundamental understanding of their functionality, properties, and performance in various applications (Figure 1), such as lignocellulosic bioplastics. Non-etheless, even these bio-based polymeric materials need to be designed to ensure sufficient recyclability and (bio)degradation.

Cellulose can be converted into various derivatives with different sizes, such as cellulose nanofibers (CNF) and nanocrystals (CNC), by different strategies. These nanomaterials with diameters ranging from tens to hundreds of nanometers exhibit high surface-area-to-volume ratio and tunable flexibility, which facilitates them suitability for a variety of applications, including energy harvesting/conversion/storage components, electronic/photonic devices, biomedical scaffolds, *etc.* Hemicelluloses comprise broad types of heteropolysaccharides such as arabinoxylan that is composed of a backbone of anhydroxylose units linked by β-1,4 glycosidic bonds, which can be designed into promising functional materials based on its special macromolecular architectures. Lignin as the most abundant aromatic polymer that holds significant prospects in constructing many emerging functional materials such as polymer blends and particle reinforced composites. Lignin has indeed become a biomacromolecule of broad interest owing to its natural functionality and unharnessed potential in developing sustainable, low-cost, and environmentally benign materials.

The Research Topic “*Lignocellulosic biomass-based materials design, fabrication, and applications*” involves the valorization of lignin, hemicelluloses, and extractives into functional materials, as well as biomass-derived material statistical analysis. Here we greatly appreciate all the contributors for their superb work on this Research Topic. Following are the highlights drawn from the contributions to this Research Topic.

In order to excavate more applicable possibilities for lignin conversion into biobased materials, Esakkimuthu et al. fabricated the lignin-poly (lactic acid) (PLA) biocomposites with an aim to improve the processability and compatibility of lignin with PLA through etherification of lignin. Kraft lignin (KL) and oxypropylated kraft lignin (OPKL) were integrated with PLA at different weight percentages (1%, 5%, 10%, 20%, and 40%) followed by injection molding. It was found that the incorporation of KL or OPKL improved the thermal stability of the composites, and the good stability of the composite in the migration test was also proved to meet the European standard even at higher lignin content of 40%. Wang et al. isolated different lignins from tobacco stalk using deep eutectic solvent (DES) extraction and alkaline delignification processes. The DES lignin possessed a higher yield and homogenous molecular structure as well as micro-nanoparticle sizes. Moreover, all the recovered lignin samples presented excellent UV absorption and structure-related absorption performance or thermal properties, which make them ideal feedstocks for producing sustainable functional or micro/nanomaterials. Besides lignin, aromatic flavonoids exhibit biological activities of interest especially in pharmaceutical applications. Park et al. prepared high content of taxifolin aglycone extract by enzymatic hydrolysis to investigate the hair tonic and hair loss inhibitors effect of taxifolin. They confirmed that a representative factor for promoting hair growth, IGF-1, was significantly increased and that TGF-β1, a representative biomarker for hair loss, was significantly reduced with taxifolin treatment.

Energy materials is another emerging area of activity within the field of lignocellulosic materials. In view of the advanced application of lignin in the electrochemical field, Li and Shi comprehensively reviewed the electrochemical energy storage application of lignin-derived carbon materials from the viewpoint of process-structure-properties-performance correlation, to highlight the valorization of biorefinery lignin in high performance energy storage materials. Suzuki et al. presented a self-standing lignin-attached graphite film that exhibits high electrical conductivity (∼2,075 S cm^−1^), which can also act as an antiplasticizer and a conductive filler for polymer films. Statistical approaches are essential tools in the analysis of lignocellulosic materials, given the multifaceted nature of variability in plant biomass. Within this stream, Pennells and Martin proposed the cross-disciplinary utilization of statistical modelling approaches commonly applied within the field of statistical genetics to evaluate data generated in the field of biomass-based material research and development. The authors reported that these statistical modelling approaches provide more depth to the investigation of biomass-processing-structure-property-performance relationships, which are not only applicable to nanocellulose production but to all biomass-based materials and products. Zhang et al. developed a template-free and one-step carbonization process to fabricate graphitic porous carbon spheres (GPCSs) from hemicelluloses and used them as the electrode material for supercapacitors. It was observed that the GPCSs exhibited a regular spherical shape, high nanoporosity, and graphitization degree. More importantly, the GPCSs electrode displayed outstanding electrochemical performance including high specific capacitance (262 F g^−1^ at 1.0 A g^−1^), rate capability energy (80%, 20 A g^−1^), and excellent cycling stability (95%, 10,000 cycles).

